# Optimization and Scale-Up for Polymer Extrusion

**DOI:** 10.3390/polym13101547

**Published:** 2021-05-12

**Authors:** Andrzej Nastaj, Krzysztof Wilczyński

**Affiliations:** Polymer Processing Department, Faculty of Production Engineering, Warsaw University of Technology, Narbutta 85, 02-524 Warsaw, Poland; a.nastaj@wip.pw.edu.pl

**Keywords:** polymers, extrusion, optimization, scale-up

## Abstract

A review paper is presented on optimization and scale-up for polymer extrusion, both single screw and twin screw extrusion. Optimization consists in obtaining a multidimensional space of process output variables (response surface) on the basis of an appropriate set of input data and searching for extreme values in this space. Scaling consists in changing the scale of the process according to specific criteria, that is, changing the process while maintaining the scaling parameters at a level that is as close to the reference process parameters as possible. It consists in minimizing the differences between the parameters characterizing the reference process and the resulting process. This may be obtained by using optimization techniques leading to the minimization of discrepancies between the parameters of scaled processes. In the paper, it was stated that optimization and scale-up based on process simulation are more effective than those based on experimentation which is time consuming and expensive. The state-of-the-art on extrusion process modeling which is the basis of optimization and scale-up has been presented. Various optimization techniques have been discussed, and the Genetic Algorithms have been identified as powerful and very efficient. Optimization and scale-up based on the process simulation using Genetic Algorithms have been broadly reviewed and discussed. It was concluded that, up to date, there is a lack of optimization studies on the counter-rotating twin screw extrusion, although the global models of this process are known. There is also a lack of process simulation-based scaling-up studies, both on the counter-rotating twin screw extrusion and on the starve fed single screw extrusion. Finally, development perspectives in this field have been discussed.

## 1. Introduction

Designing the polymer processing is nowadays aided by computer simulations which are based on the mathematical models of manufacturing processes. Computer modeling enables us to predict the process flow on the basis of process parameters (material characteristics, machine geometry, operating conditions). The process, e.g., extrusion, can be characterized by the flow rate, i.e., extrusion throughput, pressure/temperature distribution, energy consumption, mixing efficiency, etc. Thus, it is possible to predict the output process parameters based on the input parameters.

Process computer modeling, however, does not solve the inverse issue of determining the process input parameters to obtain the optimal output parameters. The models do not enable us to select the process parameters that meet the production aims, e.g., maximizing process throughput, mixing efficiency, or minimizing energy consumption. Thus, these do not enable us to optimize the process according to the specific optimization criteria.

Extrusion is the most important technology in the polymer processing industry. It is broadly used for the production of film, sheet, pipe and profiles, and for specialty operations, such as compounding, mixing, pelletizing, etc. Optimization of an extrusion is a conflicting, multi-objective issue. It is complicated by the number of data (material parameters, geometry data, operating conditions) and their non-linear relations, as well as by the opposing criteria, e.g., extrusion output and energy consumption. It is difficult to find a global process optimum avoiding local optima. 

In general, optimization consists in obtaining a multidimensional space of process output variables (response surface) on the basis of an appropriate set of input data and searching for extreme values in this space.

**Figure 1:**
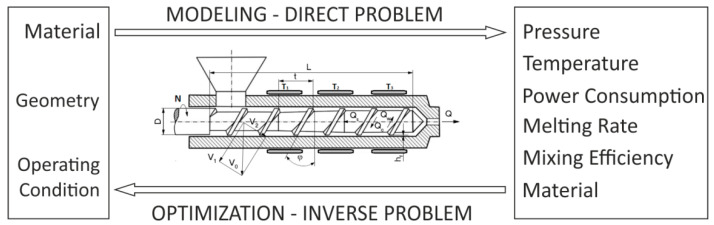
Direct (modeling) and inverse (optimization) problem for polymer extrusion: L, D, t, φ, h—screw geometry parameters, N—screw speed, Q—flow rate, T—temperature conditions, V—velocity.

Modeling (simulating) the process, that is predicting the process course, is a direct problem, while optimization which is looking for an optimal solution with respect to the optimal criteria is an inverse problem, which is schematically depicted in Figure 1.

The optimization issue can be solved by experiment or simulations. The first optimization tests were performed with experiments using various screws and operating conditions. The process optimum was simply the best one tried. This approach was time-consuming, expensive, and nothing warranted that the global optimum was found. Statistical methods, regression analysis and the response surface method may be used for optimization both by experiment and simulations. 

Underwood [1] was the first who applied the factorial design of experiments to optimize the extrusion process. The optimum was found by searching the extremum on the response surfaces relating the input and output process parameters. Later, other researches applied this approach, e.g., Verbraak and Meijer [2]. The main drawback of this approach was the number of necessary experiments. 

**Figure 2:**
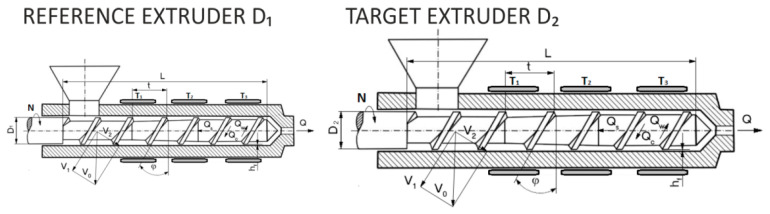
The concept of scaling-up for polymer extrusion: D_1_—reference screw diameter, D_2_—target screw diameter.

An important method of designing in the polymer processing is the process scaling, i.e., changing the scale of the process according to the specific criteria. Scaling consists in changing the process, e.g., increasing the process efficiency, while maintaining the scaling parameters at a level that is as close (similar) to the reference process parameters as possible. Thus, it consists in minimizing the differences between the parameters characterizing the reference process and the resulting process. The basis of this concept is the theory of similarity, which determines the relationships between the physical parameters that affect the phenomenon under study (e.g., fluid flow). Meeting these dependencies ensures that the similarity between two systems of different sizes is maintained. The concept of scaling-up for polymer extrusion is depicted in Figure 2.

First, extrusion scaling was performed on the basis of single-parameter scaling criteria which characterized only the selected process features. This was broadly discussed by Rauwendaal [3]. Scaling based on a computer model of the process enables us to change the scale of extrusion based on the characteristics of the entire process. Optimization techniques leading to the minimization of discrepancies between the parameters of scaled processes may be used. 

Thus, using process modeling seemed to be the fundamental and most efficient approach both for optimization and scaling-up the polymer extrusion. 

## 2. Modeling of Extrusion

In the modeling of polymer processing, the models are, in general, deterministic transport phenomena based on either steady (continuous processes, e.g., extrusion) or unsteady (cyclic processes, e.g., injection molding) processes, and contain distributed parameters or locally lumped parameters. For engineering tasks, the lumped parameter models are generally sufficient [4]. The main target of engineering designs is to predict the pressure and polymer melt temperature profiles along the machine for a given screw and die geometry as a function of the operating conditions. In these models, the screw channel is usually divided into short elements (increments), where the input data (e.g., temperature and pressure) come from the computation in the previous element. In addition, the output data from the current element are the input data for the next element. Within each element the local process parameters are assumed to be constant. 

The lumped parameter concept is particularly useful when modeling the processes with polymer plasticization, e.g., extrusion or injection molding, where besides the melt flow, we are faced with solid conveying and melting of the material.

With tremendous advances in computational fluid dynamics, the present concepts in modeling of polymer processing are towards using very sophisticated numerical methods (e.g., finite element method, FEM). These include both two- or three-dimensional calculations of the process parameters, e.g., velocity, stress, pressure and temperature fields, with a variety of boundary/initial conditions for non-Newtonian and temperature-dependent, and often viscoelastic fluids. In extrusion and injection molding, however, there are a lot of other problems which are still unsolved, and thus these sophisticated methods are currently not applied for composite (global) modeling of these processes. 

The term global modeling means modeling the interacting phenomena that occur in the screw/die system. This requires the use of a computation algorithm appropriate for a given type of extrusion. For flood fed extrusion with gravitational feeding, the forward scheme of computation is applied. In this case, the material flow rate is not known and results from the screw/die co-operation, and must be determined in multiple iterative computations. For starve fed extrusion with metered feeding, the backward scheme of computation is used, i.e., the inverse computation algorithm, since there is no continuous flow rate/pressure relation along the screw/die system. In this case, the flow rate is known and equals the flow rate of the material metered into the extruder by the feeding device. This issue was discussed in detail by Wilczyński et al. [5].

The state-of-the-art for composite modeling of screw processing (extrusion and injection molding) was presented in some fundamental books, e.g., by Tadmor and Klein [4,6], Hensen et al. [7], White and Potente [8], Rauwendaal [3], Osswald and Hernandez-Ortiz [9], and Agassant et al. [10], as well as in some review papers, e.g., by Arrifin and Ahmad [11], Wilczyński et al. [12], Teixeira et al. [13], Malik et al. [14], and recently by Wilczyński et al. [5] and Hyvärinen et al. [15]. Single screw extrusion, twin screw extrusion, both co-rotating and counter-rotating, and injection molding were considered, as well as the flood fed and starve fed operations were discussed. 

Tadmor and Klein [4] developed the first computer program EXTRUD for simulation of single screw extrusion which was described in detail in [16], and later, Klein and Klein presented the SPR simulation system [17]. Next, several other computer models and programs for single screw extrusion were developed, e.g., the NEXTRUCAD program by Agur and Vlachopoulos [18], the REX program by Potente et al. [19,20], the PASS system by Sebastian and Rakos [21], the SSEM program by Wilczyński [22,23], as well as the computer models developed by Fukase and Kunio [24], Zavadsky and Karnis [25], Vincelette et al. [26], Amellal and Lafleur [27], and Gaspar-Cunha and Covas [28,29]. Recently, Wilczyński et al. [30,31] built the computer model and program for simulating the single screw extrusion of wood plastic composites. 

Studies on the modeling of co-rotating twin screw extrusion were initiated by White and co-workers. On the basis of the melt flow modeling [32,33] and the polymer melting modeling [34,35], the computer model of co-rotating twin screw extrusion Akro-Co-Twin [36,37,38] was developed. Independent research carried out by Potente [39,40,41] led to the development of the SIGMA program [42,43], and the research made by Vergnes et al. [44,45,46], led to the development of the LUDOVIC program [47]. Canedo [48] developed the TXSTM program, and Teixeira et al. [13] built the global software for co-rotating extruders.

Studies on the modeling of counter-rotating twin screw extrusion were also initiated by White and co-workers. On the basis of the melt flow modeling [49,50] and the polymer melting modeling [51,52], the first computer model of counter-rotating twin screw extrusion Akro-Counter-Twin was developed [53,54]. This research was continued by Wilczyński et al. [55,56,57] who built the TSEM program. 

Studies on the modeling of starve fed single screw extrusion were much more limited. Wilczyński et al., based on the polymer melting modeling [58,59], developed the first, and up to now the only available computer model of this process, i.e., the SSEM-Starve program [60,61]. This model was later extended to non-conventional screw configurations [62,63], and to extrusion of polymer blends [64,65] and wood plastic composites [66,67]. Recently, the global GSEM model was developed which enables the modeling of single screw extrusion both in the flood fed and starve fed mode [68,69]. 

The starve fed extrusion has several advantages over the flood fed extrusion which was discussed in the literature, e.g., [70,71,72,73,74], and very recently in [75]. The pressure development along the screw is lower, and there is a less chance of material agglomeration, and mixing is substantially improved. Melting is faster since the granules are not compacted into a dense solid bed, and the polymer pellets maintain their individuality as the melting progresses. In the starve fed extrusion, the screw speed can be varied at the constant extruder throughput, and the extruder throughput can be varied at the constant screw speed, and this allows for a greater degree of the process control. In general, starve fed extruders may be used for more demanding processing tasks, e.g., [75], although there are also some disadvantages. The extruder throughput is reduced below its capacity, and the process operation is more complicated in that an external device is necessary to deliver the polymer into the extruder.

**Figure 3:**
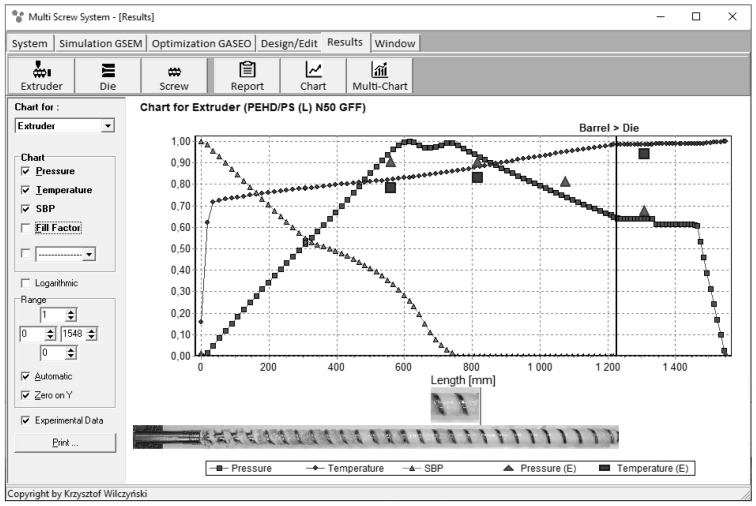
Overall extrusion process characteristics, simulation and experimentation data using the GSEM model—flood fed single screw extrusion: SBP—solid bed profile, E—experiment [68].

**Figure 4:**
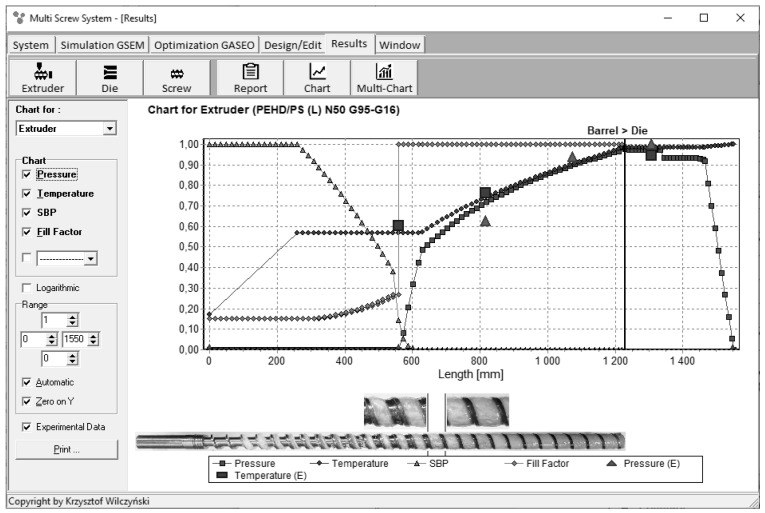
Overall extrusion characteristics, simulation and experimentation data using the GSEM model—starve fed single screw extrusion: SBP—solid bed profile, E—experiment [68].

Examples of extrusion process simulations are depicted in Figure 3 and Figure 4. These simulations were validated by experiments. The dimensionless overall process characteristics are presented which include pressure and temperature profiles, the melting profile, as well as the screw filling profile for starve fed extrusion (Figure 4). It is seen here that the pressure falls to zero when starvation begins. 

## 3. Optimization for Extrusion

In the industry, in practice, for years, the screw and extrusion process designing was accomplished on a trial-and-error basis. Nevertheless, some attempts of a scientific approach to this issue have been reported. The most complete approach has been presented by Rauwendaal [3] who discussed various design criteria, that is, screw mechanical strength, extrusion output (optimizing for melt conveying, for plasticization, and for solids conveying), and process power consumption. In this approach, the analytical equations describing each extrusion step were used to solve the designing and optimizing task. 

First trials of extrusion optimization by computer simulation were performed by Tadmor and Klein [6], who applied the regression analysis and response surface analysis for screw design by simulation using the EXTRUD program [16]. Later, Maddock and Smith [76] presented the extruder designing by using computer simulation printout. Helmy and Parnaby [77] developed a computer software package by applying a steady-state hill-climbing optimization routine to the melt extruder screw design. All these studies were limited to conventional screws.

Later, more sophisticated models were proposed. Potente et al. [78,79] proposed a strategy for screw optimization by means of DOE (Design of Experiments) and multiple regression using the extrusion model REX [19,20,80]. Thibodeau and Lafleur [81,82] used the STATISTICA program to search the optimum on a response surface given by the extrusion model of Ecole Polytechnique de Montreal [26,27]. Statistical concepts were also applied by the authors of [83] who used the SSEM program [84]. The main drawback of the statistical approach was the number of necessary simulations in the response surface of high data density, and the danger of finding local optima rather than the global one. 

Artificial Intelligence (AI), e.g., Neural Networks (NN), Genetic Algorithms (GA) and Fuzzy Systems (FS), may be also applied for modeling and optimization of manufacturing processes. These deliver continuous or discrete solutions based on the learning process with the use of available data. 

Genetic Algorithms compared to other optimization methods have the following features [85]:

- the parameters of the optimization problem are processed in coded form and not directly;
- searching for a solution is carried out from a certain population and not from one point, which ensures that the probability of getting stuck in a local extreme is low;
- the selection rules are probabilistic rather than deterministic;
- a new search area of expected higher quality is determined based on the previous experiences, thanks to which, despite some randomness, they do not amount to accidental wandering;
- only the objective function is used and not its derivatives.

In general, the goal of optimization is to find the global optimum in a given search space by maximizing or minimizing some objective function that can be subjected to some constraints. Fundamental research using Genetic Algorithms was performed by Covas and Gaspar-Cunha who first developed optimization procedures for single screw extrusion [86,87,88,89], and co-rotating twin screw extrusion [90,91,92,93]. These include two optimization routines.

In the first routine, a global objective function is defined as a simple scalar function, e.g., as the weighted sum of the individual criteria

(1) $$F_{obj}=\overset{k}{\underset{i=1}{\sum }}w_{i}f_{i\_norm}$$

where *F_obj_* is the global objective function, *f_i_norm_* is the normalized individual criterion *f_i_*, *w_i_* is the weight of the individual criterion, and $\sum^{\;}w_{i}=1$, *k* is the number of individual criteria.

The global objective function to be maximized combines the individual functions, depending on whether the corresponding criterion is to be maximized or minimized, which are represented, respectively, in the following form

(2) $$f_{i\_norm}=\frac{f_{i}-f_{min}}{f_{max}-f_{min}}$$

(3) $$f_{\;i\_norm}=\frac{f_{max}-f_{\;i}}{f_{\;max}-f_{\;min}}$$

where *f_max_*, *f_min_* represent the maximum and minimum values of *f_i_*, respectively, and refer to the process response constraints.

The optimization procedure includes:

- identifying the process variables to be optimized, and defining the range of their variation;
- selecting the optimization criteria, and prescribing their boundaries and quantifying the relative importance by weighting;
- searching the solution, maximizing the objective function.

Using this approach, various solutions to the same optimization task may be obtained if the number and weights of the individual criteria are valued differently. Moreover, this methodology lacks flexibility. If some constraints or weights are to be modified, the optimization procedure has to be repeated. Moreover, some solutions may be shadowed. 

In the second routine, the optimization of several individual criteria is considered, i.e., the solution space is seen as sets of dominated and non-dominated points. A non-dominated point is a solution at least as good as the others with respect to all objectives, but strictly better with respect to at least one objective. Therefore, one solution point dominates another when it is as good in every objective as the others with respect to all objectives and formally better in at least one objective. The set of non-dominated points is known as the Pareto optimal set. In this case, therefore, there is no need to define a priori the weights in a global objective function since selecting the solution can be seen as setting those weights. Pareto curves define the optimal range of all criteria and the corresponding compromise. 

The Genetic Algorithm procedure requires, e.g., [85]:

- encoding input variables which are represented as strings of bits, referred to as chromosomes;
- defining an objective function that evaluates a value of each genotype, i.e., a set of chromosomes;
- generation of a randomly selected initial population;
- definition of genetic operators, i.e., reproduction, crossover and mutation, which search the response space using probabilistic rules.

There are several ways of implementing the genetic operators, e.g., [79]. When utilizing the weighted global objective function, a roulette wheel selection may be used as reproduction operator, whereas in multi-objective optimization, tournament selection may be applied.

Covas et al. [87] first considered the optimization task of setting the operating conditions for a conventional single screw extruder. As input variables, the screw rotation speed and three barrel temperature profiles were assumed. The global objective function defined the number of optimizing criteria and their relative importance. The four criteria were selected as the most relevant, namely the extruder output, the melt temperature at the die inlet, the length of screw required for melting to be completed, and the power consumption.

Next, Covas and Gaspar-Cunha [88,89] considered the optimization of screw design in a single screw extruder, and applied the optimization strategies discussed above, i.e., using a global objective function and performing a multi-objective optimization based on the Pareto curves. In this study, the objective was to define the length of the feed and compression sections of the screw, the diameter of the screw root in the feed and metering sections, the screw pitch, and the flight clearance of the screw. The screw diameter and the screw length remained constant to fit the available extruder. The screw should maximize the extruder output and the degree of mixing defined by the WATS index, and minimize the length of screw required for melting, the outlet melt temperature, the power consumption, and the viscous dissipation.

In the next study, Gaspar-Cunha et al. [90,91] tested the optimization algorithms by considering the problem of setting the best operating conditions for a co-rotating twin screw extruder. Two possibilities were discussed, the maximization of an objective function, and the simultaneous optimization of the individual criteria and the use of Pareto plots. The flow conditions inside the extruder were simulated using the LUDOVIC software [47]. Various case studies were performed, among them a reactive extrusion application. As input variables, the screw rotation speed, the extruder output and three/five barrel temperature profiles were considered. The six optimization criteria were selected as the most relevant, namely the extruder output and the degree of mixing which should be maximized, and the power consumption and the average residence time to be minimized, as well as the melt temperature at the die exit which should stay within the prescribed range, and the maximum melt temperature which should be excluded from the prescribed range. It is important to note that the extruder output was both the parameter to be optimized and the optimization criterion, since maximizing it was desirable regardless of the fact that it is described by the feeder.

Furthermore, Gaspar-Cunha et al. [92,93] considered the optimization of screw design in a co-rotating twin screw extruder, using the multi-objective evolutionary algorithms. This optimization was performed in terms of the most adequate location of a pre-selected set of screw elements, in order to maximize a prescribed performance. An application to the complex case of reactive extrusion was also presented. 

It is important to notice that the problem of determining the screw geometry for co-rotating twin screw extrusion is different from that for single screw extrusion. In the latter, the main geometrical variables (that is, the channel depth in the feed zone, the channel depth in the metering zone, the length/diameter ratio of each screw zone, and the helix angle) can vary continuously within a prescribed range. In the former, the screw is determined by selecting a fixed number of individual screw elements from a wider set of elements usually available and determining their relative position along the screw. Different types of optimization, including the simultaneous optimization of the screw profile and the processing conditions were presented.

The optimization of single screw extruders and co-rotating twin-screw extruders has been summarized by Covas and Cunha [94]. They presented the process modeling routines followed by a discussion on optimization algorithms which can be successfully adopted. It was concluded that the optimization methodology proposed there is able to solve the problems with very distinct characteristics, with continuous and discrete variables, as well as with a mix of them. In the case of single extrusion, all variables are continuous, however, in the case of co-rotating twin screw extrusion, both situations can take place. The results obtained for several examples seemed to be in agreement with the current process knowledge, and the solutions received were feasible and could be implemented in extrusion practice.

Recently, the authors performed optimization studies [95,96] for the starve fed single screw extrusion. The flow conditions inside the extruder were simulated using the global models of this process [60,63]. Furthermore, a novel computer optimization system for the flood fed/starve fed single screw extrusion has been developed [68]. This coupled system enables us to optimize single screw extrusion operating both in the flood fed and/or starve fed mode. Optimization is based on the process simulation which is performed using the global extrusion model GSEM (Global Screw Extrusion Model) [68,69]. The process is optimized using the GASEO (Genetic Algorithms Screw Extrusion Optimization) procedures which are based on the Genetic Algorithms. These procedures are defined by the Genetic Algorithms parameters, the number of optimized parameters, the initial population size, the length of chromosomes, the probability of crossover, the crossover points, and the probability of mutation. Optimization can be performed selectively for flood fed extrusion or starve fed extrusion, but can also be performed in a unique way in a coupled manner when both modes of feeding are allowed.

In the study [68], the optimization task of setting the operating conditions and screw design has been presented to maximize the extrusion output and minimize the specific energy consumption defined by the power consumption/extrusion output ratio. The optimized parameters were the screw speed, the three barrel temperatures, and the length of the screw metering section. The extruder output was both the parameter to be optimized and the optimization criterion, since maximizing it was desirable regardless of the fact that it is described by the feeder. The global objective function was defined as the weighted sum of the individual criteria (Equation (1)). The values of optimized parameters for computation of the global objective function were selected randomly. The starve fed or flood fed modes of feeding were also selected randomly.

**Figure 5:**
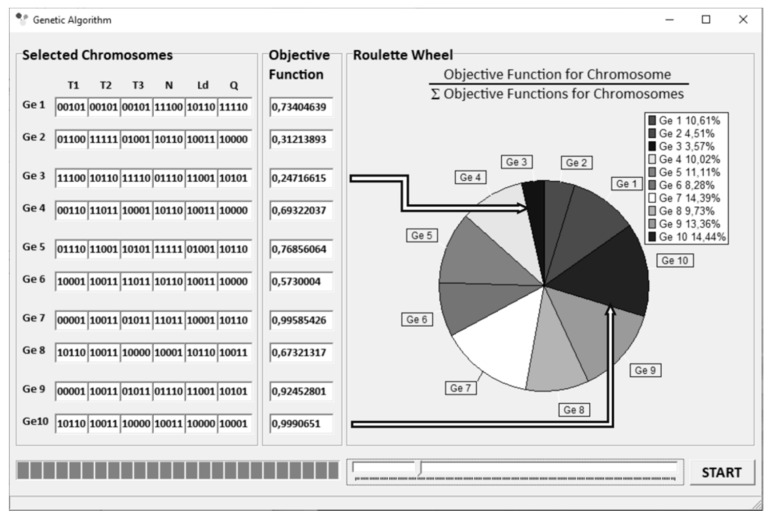
Execution of Genetic Algorithms: selection of initial population and evaluation of chromosome adaptation [68].

**Figure 6:**
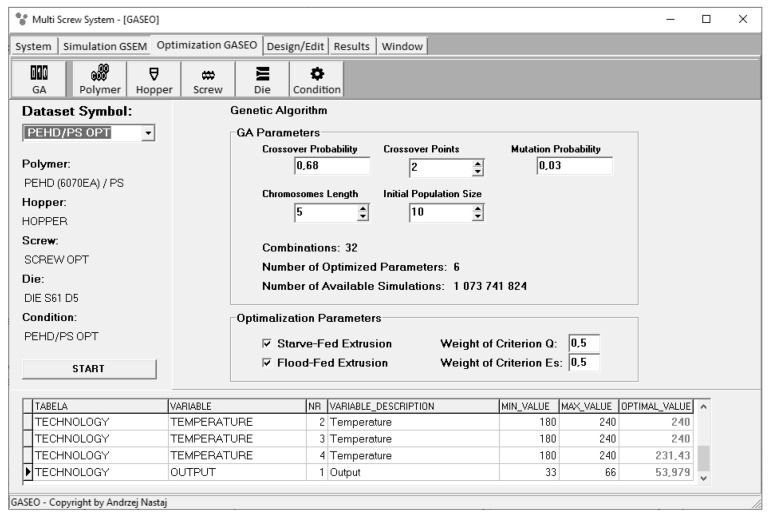
Genetic Algorithms parameters defining the optimization procedure [68].

The “roulette wheel” method was applied for selection [68]. A scheme of operation of “roulette wheel” is depicted in Figure 5. The surfaces of “roulette wheel” assigned to individual genotypes are proportional to the values of the objective functions produced by these genotypes. For example, the Ge10 genotype produces the highest value of the objective function F_obj_ = 0.9991, and covers an area on the “roulette wheel” equal to 14.44% of its total area. However, the Ge3 genotype produces the smallest value of the objective function F_obj_ = 0.2472, covering an area equal to 3.57% of the total area of the “roulette wheel”. The Genetic Algorithms parameters are depicted in Figure 6.

It is worth noting that the optimization indicated the extrusion with starving as an optimal solution. In this case, the global objective function reached the highest value. The extruder output was relatively high and the specific energy consumption was minimal.

**Figure 7:**
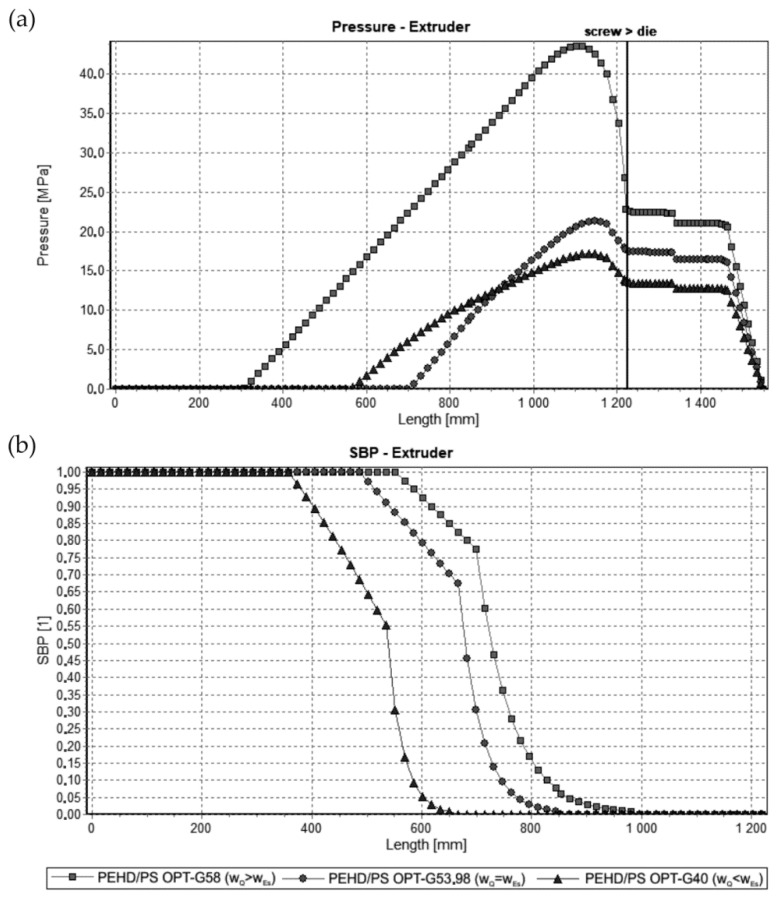
Simulations for starve fed extrusion at optimal process parameters for various weights of optimization criteria w_Q_ and w_Es_ (adapted from [68]: (**a**) pressure profiles, (**b**) melting profiles (SBP, i.e., solid bed profile).

**Figure 8:**
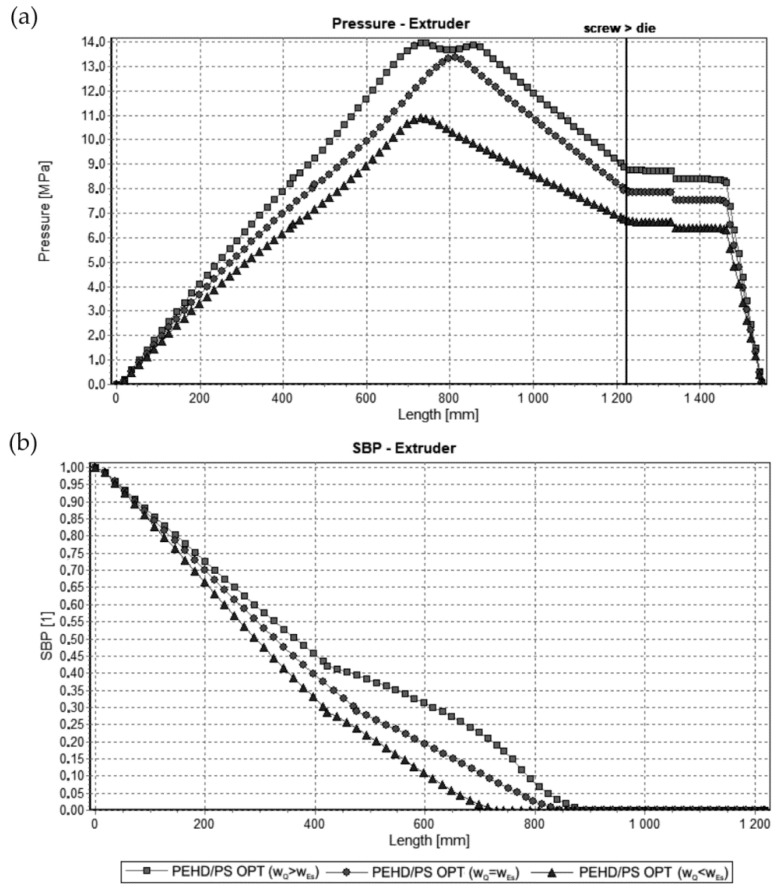
Simulations for flood fed extrusion at optimal process parameters for various weights of optimization criteria w_Q_ and w_Es_ (adapted from [68]): (**a**) pressure profiles, (**b**) melting profiles (SBP, i.e., solid bed profile).

The results of optimization are dependent on the weights of the optimization criteria assumed. This is illustrated in Figure 7 and Figure 8, where simulations at optimal process parameters for various weights of optimization criteria are depicted. Pressure profiles, depicted in Figure 7a and Figure 8a, clearly show that when the flow rate is preferred, i.e., w_Q_ > w_Es_, the pressure substantially increases, and when the specific energy consumption is preferred, i.e., w_Q_ < w_Es_, the pressure decreases. It results from Figure 7b and Figure 8b that when the flow rate is preferred, i.e., w_Q_ > w_Es_, the melting is slower, and opposite, when the specific energy consumption is preferred, i.e., w_Q_ < w_Es_, the melting is faster.

Up to date, there is a lack of optimization studies on counter-rotating twin screw extrusion, although the global models of this process have been developed [53,54,57]. It seems to be reasonable to apply the Genetic Algorithms to solve this optimization task, as in the case of optimization of co-rotating twin screw extrusion. The problem of defining the screw geometry for the counter-rotating twin screw extrusion is similar to the co-rotating twin screw extrusion, and different from that for the single screw extrusion. In the former, the screw is defined by selecting the screw elements from a set of elements usually available and defining their position along the screw. In the latter, the geometric variables can vary continuously within a prescribed range.

It seems that in the paper, which is essentially about the extrusion process, it is worth devoting a few words to the injection molding process because of its importance in the polymer processing as well as because of some similarity to the extrusion process.

Fernandes et al. [97] discussed the problem of modeling and optimization of the injection molding (IM) process in detail in a very fundamental review paper. The state of-the-art studies concerning the mathematical modeling of injection molding were presented, and the revision of the literature regarding the optimization of injection molding based on various techniques was also discussed. These optimization techniques included design of experiments, artificial neural networks, and evolutionary algorithms. The strengths and weaknesses of each approach were discussed. Finally, the optimization research performed in the injection molding regarding some of the specific problems associated with the process was reviewed, such as the runner system and the cooling channels design, the process conditions setting, the gate locating, and the cavity pressure balancing. 

The Genetic Algorithms discussed in this paper were mostly used for the optimization task of setting the operating conditions, e.g., by Mok et al. [98], Turng et al. [99,100], Ozcelik et al. [101,102,103], Gaspar-Cunha et al. [104,105,106], Shen et al. [107], Chen et al. [108,109], Wu et al. [110], Ding et al. [111], Natalini et al. [112], and Tsai and Luo [113]. The optimization of the runner system was discussed by Wu et al. [110] and Alam and Kamal [114,115]. The optimization of the gate location was considered by Young [116] and Kim et al. [117]. The optimization of the cooling was discussed by Fernandes et al. [106], Lam et al. [118], Qiao [119], and Cheng et al. [120].

Kashyap and Datta [121] presented a review of different techniques employed for optimizing various injection molding parameters along with their advantages and limitations. It was concluded in the review that a complete intelligent technique operable without human interference is yet to be developed. Very recently, Wilczyński and Narowski [122] also discussed the optimization issues of the injection molding process.

## 4. Scale-Up for Extrusion

The process scaling consists in changing the scale of the process according to the specific criteria, that is changing the process, while maintaining the scaling parameters at a level that is as close to the reference process parameters as possible. 

The scale-up of extrusion consists in defining the geometry and/or the operating conditions of a given extruder that replicate the operation of the reference extruder. This is a procedure of great engineering importance, e.g., the scale-up rules enable the design of large extruders using the results of studies performed on the laboratory scale.

Over the years, a number of various scale-up concepts have been developed. The scaling-up of extrusion was presented in some fundamental books, e.g., by Rauwendaal [3], Hensen et al. [7], McKelvey [123], Stevens and Covas [124], Campbell and Spalding [125], Chung [126], Chen et al. [127], as well as in a number of papers. 

Most scale-up concepts used the analytical process descriptions to correlate the so-called large and small primary scaling variables (that is, the screw diameter, the screw channel depth, the screw length, and the screw speed) in terms of an exponent of the ratio of the reference and target screw diameters

(4) $$\frac{X_{2}}{X_{1}}={\left(\frac{D_{2}}{D_{1}}\right)}^{x}$$

where *X*_1_ and *X*_2_ are the small and large scaling variables, respectively, *D*_1_ and *D*_2_ are the small and large diameter, and *x* is the scale up index.

The first extrusion scale-up concepts were developed by Carley and McKelvey [128] who analyzed the melt conveying in the single screw extruder, and proposed the scaling rules for geometrically similar extruders, for which the screw channel depths and widths were increased in proportion to the diameter ratio and the screw speed was constant, in the following form

(5) $$\frac{G_{2}}{G_{1}}={\left(\frac{D_{2}}{D_{1}}\right)}^{3}$$

(6) $$\frac{P_{2}}{P_{1}}={\left(\frac{D_{2}}{D_{1}}\right)}^{3}$$

Furthermore, several other scale-up rules for single screw extrusion were proposed, e.g., by Maddock [129,130], Fenner et al. [131,132], Pearson [133], Potente and Fischer [134], Schenkel [135], Chung [136], Rauwendaal [137], and Potente [138]. Scale-up of specific process aspects, such as mixing, have also been investigated, e.g., by Elemans and Meijer [139], and Wang and Manas-Zloczower [140]. Several studies were devoted to twin screw extrusion, e.g., by Menges and Feistkorn [141], Ganzeveld and Janssen [142], Nakatani [143], and Dryer et al. [144]. The scaling of mixing equipment in both aspects scaling-up and scaling-down was discussed in details by Potente [145].

Pearson [133] was the first who performed a complete extrusion scale-up analysis. In this analysis, the solids conveying, melting and melt conveying were analyzed, and the conclusions were that for the constant screw helix angle and the barrel temperature, the acceptable scale-up is obtained when the Graetz, Brinkman and Nahme numbers are kept constant in the various functional screw sections. The major advantages of this scale-up concept were the balanced solids conveying, melting and melt conveying, the constant specific energy consumption, and the power law dependence of the primary scaling variables.

Rauwendaal [3,137] performed a valuable comparative analysis of the effect of the existing scale-up rules on extrusion performance. In most cases, the unbalanced solids and melt conveying rates or an excessive viscous dissipation were predicted and the lack of generality of the scale-up rules was confirmed. 

The conclusions from this analysis were summarized by Covas and Gaspar-Cunha [146] who concluded that the current scale-up rules

- can tackle a single scaling criterion only (e.g., shear rate, melting rate, pumping rate) and only a single step of extrusion process (e.g., melting, melt conveying);
- can take into account only a few global geometric or operation parameters (screw diameter D, channel depth H, screw length L, and screw speed N);
- are based on the simplified mathematical description of extrusion process, have limited quantitative predictive capability, and do not cover the overall extrusion process;
- are rigid in terms of the scale-up criteria which means that the user cannot include a new criterion.

Therefore, more efficient scaling-up methods are needed which would be based on an accurate mathematical description of extrusion process and would allow for:

- considering simultaneously several process criteria;
- selecting single parameters (e.g., average shear rate) or functions (e.g., shear rate profile along the screw) as scale-up criteria;
- flexibility in selecting/defining the criteria.

Finally, Covas and Gaspar-Cunha concluded [146] that these requirements can be fulfilled by considering the extrusion scale-up as a multi-objective optimization task, where the goal is to define the geometry/operating parameters of the target extruder in such a way that the major performance measures of both extruders are as close as possible, that is, the scaling-up goal is to minimize the differences between the values of the selected process response parameters of the reference and the target extruders. Usually, the geometry/operating parameters of the reference extruder are known, and we are looking for these of the target extruder. Similar concepts were used to optimize the single and twin screw extrusion [86,87,88,89,90,91,92,93]. 

**Figure 9:**
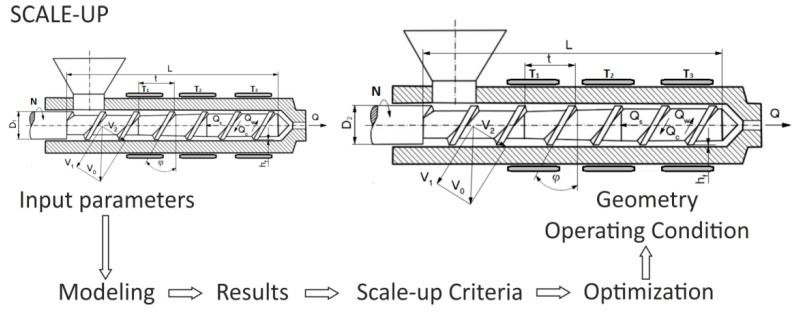
The modeling/optimization concept of scaling-up for polymer extrusion: D_1_—reference screw diameter, D_2_—target screw diameter.

The implementation of this scale-up concept requires the following actions which are illustrated in Figure 9:

- simulation of the extrusion process to obtain the responses data of the reference extruder under a specific set of geometry/operating process parameters (input parameters => modeling => results);
- defining the scaling-up criteria (results => scale-up criteria);
- specifying the fixed and known parameters of the target extruder, e.g., screw diameter D, and length/diameter ratio L/D;
- performing the scaling-up procedure by minimizing the differences between the values of the selected process response parameters of the reference and target extruders, which were defined in the second step (optimization => geometry/operating conditions).

Thus, this method includes the process modeling/simulation, defining the scaling-up criteria, and the multi-objective optimization algorithm. The scale-up criteria are computed for the target extruder on the basis of the process simulation results obtained for the input data randomly generated by the Genetic Algorithms and are compared with the equivalent ones for the reference extruder. The quality of each solution is evaluated and the searching of a good solution is iteratively repeated until a satisfying minimization of the differences is obtained. 

The appropriate selection of the scale-up criteria is fundamental for a good process scaling-up. Usually, the common scale-up factors include the shear rate, solid conveying rate, melting rate, pumping rate, residence time, power consumption, specific energy consumption, and area/throughput, as proposed by Rauwendaal [137] and Potente [138]. 

Covas and Gaspar-Cunha [146] propose using the following scale-up criteria which are not inherently related to machine size, that is:

- the ratio of the total flow rate to the drag flow rate, i.e., Q/(WHND), where Q is the flow rate, W is the screw channel with, H is the screw channel height, N is the screw speed, and D is the screw diameter;
- the specific mechanical energy, defined by the energy consumption per unit output;
- the pressure change over the unit channel, i.e., ΔpL/H, where Δp is the pressure change, L is the screw length, and H is the screw channel height;
- the relative melting length, i.e., L_m_/L, where L_m_ is the screw length required for melting and L is the screw length;
- the average shear rate/shear stress;
- the overall viscous dissipation, i.e., T_avg_/T_b_ or T_maz_/T_b_, where T_avg_ is the average melt temperature, T_maz_ is the maximum melt temperature, and T_b_ is the barrel temperature;
- the average residence time;
- the WATS index which is the measure of the mixing degree [147].

The dimensionless similarity numbers, e.g., the Cameron, Peclet, Brinkman, and Nahme numbers may be also considered as the scale-up criteria to estimate the intensity of the thermo-mechanical effects inside the extruder, which was proposed, e.g., by Pearson [133]. The Cameron and Peclet numbers estimate the convection in the flow direction against the conduction in the cross-channel and in the flow directions, respectively, and provide information whether the flow is adiabatic or the thermal regime is fully developed. The Brinkman and Nahme numbers evaluate the importance of viscous dissipation. 

Most of the scale-up criteria can be taken as a single global value but, in some cases, it might make sense to attempt to reproduce the evolution along the screw length. Examples would be the melting profile, i.e., the solid bed profile (SBP) defined by the ratio of the solid bed width to the screw channel width (X/W), and the cumulative residence time profile.

Each of these criteria may be considered as an objective function *F_i_* to be minimized, for the single values or the functional dependencies (e.g., axial profiles), respectively, in the form [146]:

(7) $$F_{i}=\frac{\left|C_{i}-C_{i}^{r}\right|}{C_{i}^{r}}$$

(8) $$F_{i}=\frac{\sum_{k-1}^{K}\frac{\left|C_{i,k}-C_{i,k}^{r}\right|}{C_{i,k}^{r}}}{K}$$

where *F_i_* is the fitness of the *i*-criterion, $C_{i}$ and $C_{i}^{r}$ are the single values of the *i*-criterion for the target and reference extruders, respectively, and $C_{i,k}\;$and $C_{i,k}^{r}$ are the values of the *i*-criterion on the *k*-location (along the extruder) for the target and reference extruders, respectively.

A simple way to perform a multi-objective optimization is to consider a global objective function that includes all individual objectives, using, e.g., a simple scalar function in the form of Equation (1). This was discussed by Covas and Gaspar-Cunha [86,87,88,89,90,91,92,93] when dealing with process optimization.

Using this optimization approach, Covas and Gaspar-Cunha [146] performed the scaling for the single screw extrusion process in terms of operating conditions and the screw geometry, and the full scale-up in terms of both screw geometry and operating conditions. This approach enables us to consider simultaneously various criteria and take into account their relative importance. It was demonstrated that the multi-criteria scaling-up is more efficient than the scaling-up based on the single process response, since the optimization algorithm finds the solutions that satisfy various criteria simultaneously. 

Recently, Berzin et al. [148] discussed the problem of scaling-up the twin screw (reactive) extrusion. It was concluded that the usual scale-up rules based on the ratios of diameters (Equation (4)) are ineffective to a certain extent as soon as complex phenomena, like the development of a chemical reaction, are involved in the extrusion process. Estimates of these quantities by more or less accurate expressions allow to derive values for the exponents, and thus appropriate scale-up rules. However, these expressions are rough approximations which cannot address the complexity of a highly non-isothermal process with evolutionary rheological behavior. Therefore, the use of numerical models to solve these complex issues is becoming more and more common, e.g., presented by Markarian [149], Ortiz-Rodriguez and Tzoganakis [150], Pradel et al [151]. They can simply be considered through a design of experiments, but the use of optimization methods, such as those presented previously, including the Multi-Objective Evolutionary Algorithms (MOEA), have also been proposed for solving scale-up problems by Covas and Gaspar-Cunha [146,152,153,154].

## 5. Future Perspectives

Optimization and scale-up of polymer extrusion based on the process simulation using Genetic Algorithms have been recognized as very useful and effective. However, these are limited to conventional flood fed extrusion and co-rotating twin screw extrusion. Up to date, there is a lack of optimization studies on the counter-rotating twin screw extrusion, although the global models of this process are known. There is also a lack of process simulation-based scaling-up studies both on the counter-rotating twin screw extrusion and starve fed single screw extrusion. Thus, this gap should be filled.

Moreover, the following five main directions of development of the extrusion optimization and scaling-up can be distinguished:

- improvement of the models of extrusion processes which are the basis for optimization/scale-up procedures, and which could be based on the very promising concept of the coupled (DEM/CFD) modeling using the Discrete Element Method (DEM) for modeling of solid conveying and Computational Fluid Dynamics (CFD) for modeling of melting/melt flow [5];
- coupling the models of starve fed extrusion and flood fed extrusion which would allow a smooth transition between them;
- extending the use of optimization/scale-up procedures to more demanding extrusion processes, e.g., extrusion of polymer blends, composites, filled polymers, reactive extrusions, and others;
- improvement of optimization procedures which was recently discussed in the literature [154];
- extending the use of optimization/scale-up procedures to food processing [155], as well as to pharmaceutical industry [156,157], and 3D printing [158].

## 6. Conclusions

The problem of optimization and scaling-up for polymer extrusion has been presented, both for single screw and twin screw extrusion. It was concluded that the process modeling is a fundamental and very efficient method as a source of process data both for optimization and scaling-up the polymer extrusion. Optimization consists in obtaining a multidimensional space of process output variables (response surface) on the basis of an appropriate set of input data and searching for extreme values in this space. Scaling consists in changing the scale of the process according to specific criteria, that is, changing the process while maintaining the scaling parameters at a level that is as close to the reference process parameters as possible. It consists in minimizing the differences between the parameters characterizing the reference process and the resulting process. It was concluded that this target may be obtained by using optimization techniques leading to the minimization of discrepancies between the parameters of scaled processes. Optimization and scaling-up the extrusion process based on the process simulation using the Genetic Algorithms were broadly reviewed and discussed.

It was concluded that, up to date, there is a lack of optimization studies on the counter-rotating twin screw extrusion, although the global models of this process are known. It seems to be reasonable to apply the Genetic Algorithms to solve this optimization task, as in the case of optimization of the co-rotating twin screw extrusion. The problem of defining the screw geometry for the counter-rotating twin screw extrusion is similar to that for the co-rotating process, and different from that for the single screw extrusion. In the former, the screw is defined by selecting the screw elements from a set of elements usually available and defining their position along the screw. In the latter, the geometric variables can vary continuously within a prescribed range.

A novel computer optimization system for the flood fed/starve fed single screw extrusion of polymeric materials has been recently developed, and this coupled system allows to optimize single screw extrusion both flood fed and starve fed. 

So far, there is a lack of scaling-up studies both on the counter-rotating twin screw extrusion and on the starve fed single screw extrusion. To achieve this goal, it seems reasonable to apply the concept of using the optimization techniques based on the Genetic Algorithms to the minimization of discrepancies between the parameters of scaled processes, as in the case of scaling-up the conventional flood fed single screw extrusion and the co-rotating twin screw extrusion.

This methodology can be easily extended to the other polymer processing technologies, as long as sufficiently precise modeling routines are available.

## Data Availability

The data presented in this study are available on request from the corresponding author.
